# Health as Experience: Exploring Health in Daily Life Drawing From the Work of Aaron Antonovsky and John Dewey

**DOI:** 10.1177/1049732320907585

**Published:** 2020-03-14

**Authors:** Ninitha Maivorsdotter, Joacim Andersson

**Affiliations:** 1 University of Skövde, Skövde, Sweden; 2 Örebro University, Örebro, Sweden

**Keywords:** salutogenic approach, qualitative social inquiry, narrative method, life experience, health practices, qualitative method

## Abstract

Research has pursued salutogenic and narrative approaches to deal with questions about how everyday settings are constitutive for different health practices. Healthy behavior is not a distinguishable action, but a chain of activities, often embedded in other social practices. In this article, we have endeavored to describe such a chain of activities guided by the salutogenic claim of exploring the good living argued by McCuaig and Quennerstedt. We use biographical material written by Karl Ove Knausgaard who has created a life story entitled *My Struggle*. The novel is selected upon an approach influenced by Brinkmann who stresses that literature can be seen as a qualitative social inquiry in which the novelist is an expert in transforming personal life experiences into common human expressions of life. The study illustrates how research with a broader notion of health can convey experiences of health, thereby complementing (and sometimes challenging) public health evidence.

## Introduction

There are well-known risks associated with the dominance of quantitative approaches in the health sciences (Fernández-Guerrero et al., 2014). At the same time, given scholars’ efforts to re-understand and problematize the relation between people’s daily life on one hand, and health on the other (McCuaig & Quennerstedt, 2018), we are in need to develop theoretical and methodological frameworks capable of advancing qualitative research (Morse, 2002; Shaw, 2016). Two emerging fields, although not homogeneously evolving, are noticeable in this progression of a broader notion of health. One is the salutogenic tradition of health research inspired by Aaron Antonovsky (1979) that emphasizes “what creates health rather than only what are the limitations and causes of disease” (p. 12). Another is the body of research that explores health through narrative methods put forward by scholars such as David E. Polkinghorne (1988) and Barbara Czarniawski (2004). Here, multiple theoretical lenses, a variety of methodological approaches and a wide range of voices revolve around an interest in biographical particulars as narrated by the one who lives them (Chase, 2005). It is in the intersection of these emerging fields that embark on broader notions of health that this article seeks to contribute.

We have witnessed considerable empirical testing in the field of salutogenic research and know, for example, that SOC (sense of coherence) changes over a lifetime (Trap et al., 2015), that a mother’s SOC has an effect on a child’s SOC (Togari et al., 2011), and that suffering can be a source of significant learning (Oliveira, 2014). Such studies represent important contributions to an emerging field, yet few conceptual models and frameworks have been developed that further inform empirical research (Super et al., 2015) and the salutogenic construct. In the early 2000s, scholars like Suominen and Lindstrom (2008) contended that less attention had been paid to the structures in sociocultural contexts that support health than those focusing on addressing specific risk factors. This is still the case a decade later. In our review of the literature, we have identified two categories of research, including a salutogenic approach and narrative methodologies, namely, “inhabiting different spaces” (see Caine, 2010; Korpela et al., 2010; Oliffe et al., 2010) and “health practices” (see Hall, 2011; Lewis et al., 2015; van Woerkum & Bouwman, 2012). Both these categories include studies that embrace a broader notion of health, although they also seem to take a symptom, disease, or some kind of “risky” position as a starting point for the exploration of experience and therefore shed more light on “the limitations and causes of disease” rather than “what creates health.”

Drawing from this backdrop, we suggest that an important research focus is to study how distinguished events in life shape and condition people’s health practices (e.g., Kotliar, 2016; Vindrola-Padros & Johnson, 2014) and how health is situated in the context of everyday life (e.g., Pedersen et al., 2019). Further important observations evolving from this research field are that living with a disease requires more than a medical approach to symptoms (Lewis et al., 2015) and that healthy behavior is not a distinguishable action, but is often a chain of activities embedded in other social practices (van Woerkum & Bouwman, 2012). Connecting to the advancements of qualitative health research elaborated on above, this article focuses on biographical particulars as they are narrated by the person experiencing them (e.g., Chase, 2005) and introduces and illustrates a method that can be used on autobiographical material to analyze how everyday settings are constitutive of different habits and health practices over time. Grounding this method in a Dewey-informed salutogenic framework, the article not only recognizes health behavior as a chain of activities but also tries to analytically depict the transactional relationships between a person’s roles, experienced obstacles, and habits.

Against the background of salutogenic and narrative approaches, three principles are pertinent to how this method is employed. First, we need to capture ordinary living over time in all its complexity. Here, we use biographical material written by the author Karl Ove Knausgaard. Knausgaard has created a life story in six volumes entitled *My Struggle*. In this novel, the author writes about his everyday life in “real time” and includes flashbacks from earlier life to frame his story. According to sources such as *The New Yorker* (Wood, 2012), the novel is seen as an important literary masterpiece. Second, we need to analyze life experiences in all their tangible shapes and forms without reducing them to dominant health discourses. In line with Brinkmann (2009), we argue that literature can be seen as a qualitative social inquiry in which the novelist is an expert in transforming personal life experiences into common human expressions of life. Third, following the first and second principles, there is still a substantial risk of engaging in a relativization of health that reduces *life* experience to *health* experience. To avoid this, the claim is that when people talk about their lives, they also make meaning of health. Therefore, it is important to position physiological health and social health at the same analytical level.

To analyze health as experience, we use the extended biographical narration in *My Struggle* and rely on a schedule of salutogenic inspired research questions outlined by McCuaig and Quennerstedt (2018). In line with McCuaig and Quennerstedt, we argue that “a good life,” in contrast to the strong biomechanical connotations of “health,” offers a broader notion of health and a broader reflection on the subject. This supports guidance about the empirical feasibility of uncovering reciprocal relationships between different mechanisms and how they may or may not contribute to individuals’ health in their lives as a whole. Our first aim is to explore how meanings of living “a good life” are made in terms of health experience in *My Struggle.* To achieve this, the research questions given below provide us with a technique that facilitates a move beyond the privileged biomedical perspectives that often dominate health discourses in society:

**Research Question 1:** What are the components of a good life?
**Research Question 2:** What aspects of living do individuals problematize in their efforts to lead a good life?
**Research Question 3:** How are people enticed, encouraged, or co-opted into particular practices of healthy/good living?
**Research Question 4:** What health resources do people draw upon to live a good life and solve daily life challenges?

In phrasing these research questions in terms of generalized “people,” we refer to the cultural context and position through which Knausgaard is able to express himself as an author, that is, the social background he shares with his readers. Knausgaard can only write a novel that fits with his own experience and has to trust that meaning is transacted in a meaningful way through the experiences he shares with his readers (Rosenblatt, 2005). For example, people’s reading of Shakespeare’s *Hamlet* in different parts of the world and at different times in history illustrates that it is the readers’ social backgrounds that reflect differences in how they adopt/connect to literature, rather than their different capacities to understand strong experiences, such as the death of a parent. Based on the answers to these research questions, our second aim is to discuss “the goals and objectives underpinning a specific regime of/for good living” (McCuaig & Quennerstedt, 2018, p. 119).

## Conceptual Framework

In connection to the overall aim to explore how people experience and make meaning of health in everyday life, we suggest a framework that, in combining Aaron Antonovsky’s salutogenic approach to health with John Dewey’s concept of experience and habits, approaches health analyses in a nondualistic way. In this context, meaning-making refers to how experience and habits contribute to people’s ability to create microenvironments in which they are capable of realizing their interests. In the active process of microenvironment creation, people incorporate some surrounding conditions while disregarding others. This concept of environing (Andersson et al., 2018) is here described through Antonovsky’s famous river metaphor.

Antonovsky (1979) stresses health as a process stretching between the continuum of health ease and health dis-ease. A salutogenic approach implies that everyone is in some way always healthy, in contrast to the pathogenic paradigm where people are healthy or not healthy. This rejection of a healthy–unhealthy dualism is in line with Dewey’s transactional view of experience and habit. Dewey (1920/1986) uses his concept of experience to explain how people are connected to and part of the world. For him, the fact that people face the consequences of their own actions means a close connection between doing and suffering, in which both people and situations are continually transformed. That is, we are not simply witnessing sealed subjects or objects bumping into each other, but a *transaction* between an internal and external environment, in which each is connected to the other in particular ways. Just as individual organisms are taking the external environment into their internal being through processes of breathing, eating, and drinking, so too are people taking a particular health practice into their orientations and habits (Shilling, 2018).

In the field of public health, the World Health Organization (WHO) seminars are of great importance, and the seminar in 1992 is of particular interest for this article. Here, Antonovsky presents the river metaphor to illustrate his vision of salutogenesis as an approach to guide health promotion. McCuaig and Quennerstedt (2018, p. 2) summarize the seminal presentation:

Antonovsky (1996) considers curative medicine’s preoccu-pation with saving swimmers from drowning downstream, and preventive medicine’s concern with preventing people from falling or being pushed into the river upstream. Antonovsky argued, however, that from a salutogenic perspective, nobody is actually on the shore, “we are all, always, in the dangerous river of life. The twin question is: How dangerous is our river? How well can we swim?” (Antonovsky, 1996, p. 14). Accordingly, from this perspective health should always be attended to as a dynamic ever-present relation *between* the swimmer and the water.

McCuaig and Quennerstedt (2018) continue by arguing that contemporary public health research that draws on Antonovsky’s work almost exclusively focuses on the swimmer by attempting to measure the swimming ability of the person, instead of exploring “what swimming is?,” “how we learn to swim?,” and “what are the conditions of the water in which swimmers are immersed?” These are all questions that take the dynamic relation between the swimmer and the river as a starting point and align well with Dewey’s transactional approach to experience, habits, and meaning-making (Garrison, 2001, 2003).

According to Dewey (1925/1981), meaning is not to be found in the world itself, nor is it captured inside the head of an individual, but located in the practices that people are involved in. For example, Knausgaard’s habits of taking care of his children harmonize, or disharmonize, with the practice of taking care of children in the Western culture surrounding him. From this perspective, meaning is not an invisible mental structure, but a process of making, in which people respond to a social practice through action and undergo its consequences. This means that we, as scholars, are potentially able to capture people’s health practices in overt habits when they are busy living their lives and trying to inhabit spaces and make meaning of their experiences.

Dewey (1922/1983, p. 16) explains that “All virtues and vices are habits which incorporate objective forces.” Using Antonovsky’s metaphor, swimming is a number of habits incorporating the conditions of the river, at the same time as no habit can incorporate the entire conditions of the river. This means that swimmers need to discriminate among things and selectively attend to some feelings, interests, and problems while disregarding others. Antonovsky (1996) makes a similar description when he explains that the SOC construct refers to a position where “the stimuli bombarding one from the inner and outer environments were perceived as information rather than as noise” (p. 15). In our study, that distinction between “information” and “noise” implies an important analytical distinction between environment and surrounding noted by Andersson et al. (2018), namely, that anything surrounding an organism that does not enter into its functioning is not part of its environment. It is not possible to stop and deduce the meaning of each single thing we encounter before we act; that would tear and tatter the habits that keep us afloat. From a Dewey-informed salutogenic perspective, habits, then, are the tools by which we discriminate and coordinate the continuous flow of experiences into functional wholes (e.g., swimming techniques; Garrison, 2001, 2003). When swimming in the river, the swimmer draws upon resources to make sense of his or her life situation, to enact to his or her life, and to develop health. Antonovsky (1979) describes the resources used by the swimmer as diverse individual and sociocultural factors. They are something that help people avoid and dissolve stressors in terms of sociocultural challenges and demands of daily living. Contextualized in the health practice, we have identified in *My Struggle* such habits that emanate from Knausgaard’s growing recognition of what to narrate about to functionally coordinate various experiences into satisfactory swimming techniques that bind together different life events into a whole (cf. SOC). It is to elicit this environing *as an active process* (Andersson et al., 2018) that we pose our analytical questions: (a) How does Knausgaard represent a good life in the novel? (b) What are his concerns in life? (c) What factors motivate him to engage in particular practices or life activities? Finally, (d) what health resources does he use or have access to in order to live a good life and solve daily life challenges? We consider a Dewey-inspired salutogenic analysis of *My Struggle* as a unique possibility to closely follow a person who is occupied with the details of his engagement in certain places, practices, identity projects and interpersonal relationships, and ends up with a narrated version of his life events that hang together in a certain way. This claimed narrative approach and the analytical questions mean that we aim at salutogenic descriptions of the meaning-making processes of life events, rather than a measurement of certain persons’ SOC strengths.

## Method

When creating a narrative study on a biographical material such as Knausgaard’s *My Struggle* (in total around 3,500 pages), a particular interest is a focus on validation and evaluation. On one hand, the empirical material is published and available to the public. Hence, our interpretations of the novel can be evaluated by other readers. On the other hand, the material is overwhelming in its volume. Furthermore, Creswell (2013) puts forward that an important criterion for a “good” narrative study is that it focuses on a single individual (or two or three individuals). The novel fulfills this aspect of quality. Even if there are many pages to analyze, the focus is still on one individual holding the number of experiences in the novel together. Another criterion for quality in narrative research is raised by Brinkmann (2009) who stresses that a novelist can be seen as a researcher exploring human experiences using sophisticated methods; Brinkmann’s claim has guided the methodology in our study.

Furthermore, during the entire analytical process, we used a deliberative strategy (Goodyear et al., 2019) based on the work of Tracy (2010). The analytical questions were used by the two researchers independently. In every step, each researcher formulated themes, categories, and narratives (“tiny tales”) that then became the basis for deliberations about whether the themes/categories/tales had something “in common.” To deal with our own bias, Tracy’s (2010) concept of “sincerity” was used with a focus on self-reflexivity and transparency. As a research team we represent different sexes (a woman and a man), different ages (born in the 1960s and the 1980s) and different sociocultural backgrounds (working class and middle class) and during the analytical process we tried to identify our preunderstandings and “taken for granted” approaches. Different interpretations of the novel were seen as opportunities to discuss our individual preunderstandings. The deliberative strategy was used for over a year with several readings of the novel and repeated data analyses.

### The First Analytical Step

The first obvious analytical question regards how to reduce an overwhelming amount of biographical material and create data. Following Andersson and Maivorsdotter (2017), our suggestion is to start in a main narrative that is consistent with the story as we encounter it as ordinary readers of the novel. The main narrative and later subcategories must be derived from the novel in such a way that an everyday consumer of the original text will recognize it. Based on these analytical principles, we have identified two main themes, *paternity* (A) and *belonging* (B), which are explicitly narrated in all the six volumes. Knausgaard (2014a, p. 628) also refers to these two themes in the novel: “A life is simple to understand, the elements that determine it are few. In mine there were two. My father and the fact that I had never belonged anywhere.” To find the quotations, they will be referred to as (volume:page), and the author of the novel will be named K.O.

### The Second Analytical Step

Against the background of the two main themes, the text was analyzed using the analytical questions to create data. The analytical questions (mentioned above) were derived from McCuaig and Quennerstedt (2018) and have generated quote collections through which we have created subcategories to the two identified main themes. Based on this second analytical step, our reading resulted in the following subcategories: Paternity is expressed through the relations that Knausgaard makes with *fatherhood* (A1), *to fear the father* (A2), and to *drinking* (A3). The second main theme, belonging, is expressed through the relations Knausgaard makes to *authorship* (B1), *to socialize* (B2), and to *smoking* (B3). Table 1 shows which research questions (Column 1) are operationalized into analytical questions (Column 2) and which quotes selection we use to illustrate each subcategory (Column 3). Note that the subcategories were created after the data were created and that each subcategory includes data from all of the analytical questions.

**Table 1.** Second Analytical Step.

Research Questions	Analytical Questions	Selected Data Quotes That Illustrate the Subcategories (The Original Selection of Quotes Was Larger)
What are the components of a good life?	How does K.O. define a good life in the novel?	A1: (1:29) (1:33–34) (1:39) (2:319) (3:282)
		A2: (1:112–113) (2:508–509) (3:230) (6:373)
		A3: (1:74–77) (4:114–115) (4:356–357)
		B1: (1:366–367) (2:215–216) (2:501–502) (3:304–309) (4:59–62) (5:82) (5:123–125) (6:913–914) (6:976)
		B2: (4:63–65) (4:231–232) (4:242) (5:70–71) (5:556–558) (6:223–224) (6:240–241) (6:247–248)
		B3: (2:79) (6:83)
What aspects of living do individuals problematize in their efforts to live a good life?	What are K.O.’s concerns in life?	A1: (1:32–39) (3:158–159) (3:310–312) (4:179–180) (6:893–894) (6:897) (6:963)
		A2: (1:18–27) (1:44–45) (1:347–348) (1:481–482) (2:338–339) (3:38–41) (3:287) (6:92) (6:59) (6:371–372) (6:938)
		A3: (1:58) (1:192–206) (2:187) (2:221–223) (2:236) (2:298–301) (4:179–180) (4:321–322) (4:402–403) (6:355) (6:932)
		B1: (1:217–218) (2:267–270) (2:387) (2:556–557) (2:604–606) (3.395–398) (4:30) (4:462–465) (4:506) (5:65) (5:158–160) (5:188) (5:241–243) (5:262–264) (5:325–327) (5:435–436) (6:337) (6:858–859)
		B2: (1:297) (1:364–366) (1:27–28) (2:149–150) (2:348) (3:7) (3:63–66) (3:105–107) (3:221–224) (3:241–242) (3:247–248) (3:276) (3:383) (3:392) (3:443–445) (3:467–470) (4:85–86) (4:265) (4:392–393) (4:473–474) (5:76) (5:354–355) (5:485–486) (5:580–582) (6:972–973) (6:885)
		B3: (2:11) (3:321) (3:454–455) (6:911–912)
How are people enticed, encouraged, or co–opted into particular practices of healthy/good living?	What factors motivate K.O. to engage in particular practices or activities in life?	A1: (2:3–7) (2:7–19) (6:885)
		A2: (1:12–14) (1:44–45) (1:301–303) (3:119–120)
		A3: (1:27–28) (1:443–444) (2:287–289) (5:417) (6:365–366)
		B1: (1:481–482) (5:237–239)
		B2: (2:218–219) (3:105–107) (3:346) (4:103–108) (5:65) (5:384–385)
		B3: (1:48) (1:211–212) (2:72–73) (2:344–345) (5:608) (6:129–130) (6:854–855)
What health resources do people draw upon to live a good life and solve daily life challenges?	What resources does K.O. use or have access to in order to live a good life and solve daily life challenges?	A1: (2:428)
		A2: (1:12–14) (1:398) (3:50–51)
		A3: (2:210) (4:231–232)
		B1: (4:428–429) (5:55–56) (5:70–71) (5:608) (6:65)
		B2: (2:218–219) (3:105–107) (3:346) (4:103–108) (5:65) (5:384–385)
		B3: (4:189) (6:349)

### The Third and Fourth Analytical Steps

After putting these questions to the text as a strict analytical technique to create data, we used our Dewey-informed salutogenic lens to interpret the data at a more general level. The third step facilitated a creation of “tiny tales” connected to each analytical question in each subcategory. Thereafter, a narrative was created in each subcategory by sampling and structuring the *tiny tales* with the support of Dewey’s theory of meaning-making (cf. “environing”). Our analytical steps can then be described in the following way:

1. Approaching the text on the basis of the main themes in the novel (recognizable by a common reader and expressed by the author).
2. Using analytical questions to generate data and thereafter thematize data into subcategories (includes a first selection of quotes).
3. Creating “tiny tales” in connection to the data derived from each analytical question (includes a second selection/reduction of quotes) in each subcategory.
4. Using a pragmatic theory of meaning-making to sample and structure the “tiny tales” into a coherent description in each subcategory.

Table 2 shows how we have specified the analytical questions in each subcategory, which quotes have been selected to interpret Knausgaard’s meaning-making, and a short description of the crucial topics (“tiny tales”) in his different life situations.

**Table 2.** Third Analytical Step (Exemplified Through the Subcategory Fathership).

Analytical Questions	Selected Data	Description of the Crucial Topics (Tiny Tales)
How does K.O. define a good life in relation to being a father?	(1:29) (1:33–34) (1:39) (2:319) (3:282)	The importance of the balance between closeness and distance to his children.
What concerns in life are most apparent for K.O. in relation to fathership?	(1:32–39) (3:158–159) (3:310–312) (4:179–180) (6:897) (6:893–894) (6:963)	He wants his fathership to involve strong feelings (love, anger, shame, and guilt), but struggles with feeling of blame and guilt every time he becomes angry or disengaged with his children. He strives not to be the eternal son, but a father with distinct boundaries for himself as well as for his children.
What factors motivate K.O. to engage in improving his fathership	(2:3–7) (2:7–19) (6:885)	The norms of being a parent and the memories of being a child. A strong belief in the importance of children not being subordinated to adults.
What resources does K.O. use or has access to in order to solve daily life challenges according to fathership?	(2:428)	The help from persons being close to him and the aid of public institutions, as well as his personal virtues of being stoical and his capacity to endure.

The presentation of the findings in the next section is the ordered and extended descriptions that resulted from the fourth analytical step. In writing the findings, we used all the tables (one for each subcategory) that were created in the third analytical step. To navigate in the selection of quotes, the tables created in the second analytical step were also used.

## Findings

The two main themes are here presented. First, *paternity*, which consists of the subcategories *fatherhood, to fear the father*, and *drinking*, and second *belonging*, which consists of the subcategories *authorship, to socialize*, and *smoking*. Quotations are referred to as (volume:page) and the author is named K.O. (hereafter referred to as K.O.). Note that all references are to the English publication of the six volumes. Connecting to Antonovsky’s river metaphor, we regard each subcategory as a certain “swimming technique” and present them as “tiny tales” revolving around the ideals, problems, means, and resources K.O. uses in his environing.

### Paternity

In many ways, *My Struggle* is K.O.’s extended inquiry into who his father was and how K.O. has become the person he has. In the following quote, we note one of the life-changing moments and the strong experiences of interpersonal relationships that continue to occupy K.O. and become the source of his struggle:

The first time I realised what I was writing really was something, not just me wanting to be someone, or pretending to be, was when I wrote a passage about dad and started crying while I was writing. I had never done that before, never even been close. I wrote about dad and the tears were streaming down my cheeks, I could barely see the keyboard or the screen. I just hammered away. Of the existence of the grief inside me that had been released at that moment I had known nothing. (1:481–482)

K.O.’s encounter with these strong feelings and the fact that he learned to suppress them are also connected to the absence of physical touch through his childhood. For example, he remembers his grandmother as the only adult who gave hugs (3:158–159) and his very discomforting feelings when seeing his father naked (4:332). Through such feelings, he realizes a lack of closeness in his childhood and struggles with his own ideals about parent–child relations.

#### Fatherhood

The ideal of a good life in relation to being a father is to rest in an identity where the closeness and the distance to his children are in balance. In K.O.’s story, the difficulty to stay in balance is described in relation to his 2-year-old daughter and her tendency to become angry (1:33–34). As a father, he needs to become a person who is capable of strong feelings and able to handle the shame and guilt of being angry and disengaged. Connected to this ideal of a healthy balance between closeness and distance and the readiness to handle feelings of guilt in his fatherhood is also the ideal not to forsake other aesthetic values of life and surrender to the love of his children as the only meaning in life (1:39).

In the context of K.O.’s ideal of what it means to be a good father, the most intriguing problem is how to unchain himself from the burden of being the eternal son. That is, he feels particularly guilty about the probability that he may pass on his own behavior as a son to his own children (6:893). What K.O. expresses here is a desire to be a father with distinct boundaries for himself as well as for his children; his and his wife’s (Linda) adult problems and concerns must never infect the lives of the children, though he realizes that children possess a great ability to interpret their closest environment and adapt to all the subtleness of human behavior (6:893–894). His problem is that to establish a healthy relation to his children not only does he need to control himself and his temper, but he must also inhabit a totally different *being*. Parent duties are bestowed with meanings through his memories of being a child and he finds a strong belief in the importance of children not being subordinated adults (3:282). And again, “not being afraid” very much entails that he as a father is capable of handling his own balance between closeness and distance and that he works with himself on a deeper relational level (6:897).

To summarize, K.O. appears as quite alone in his ontological struggle and in his personal virtues of endurance and being stoical. In contrast, he often underlines the practical help supplied by kindergarten and his and Linda’s mothers as important resources for finding a balance between K.O. the father and K.O. the writer.

#### To fear the father

K.O.’s fear of the father is almost like a watermark stamped on every single page through the volumes. It is an anoetic feeling connected to almost any indeterminate situation he finds himself in. He sums it up in the following way:

The sole really unpredictable factor in this life, from autumn to winter, spring to summer, from one school year to the next, was dad. I was so frightened . . . (3:287)

Singling out *to fear the father* as a distinct subcategory and a concern in itself shows that K.O.’s ideal is to be at peace with who the father was and what became his destiny, one of the situations in which this is most apparent is soon after the father has died and K.O. talks to the priest about the funeral. It had been like a confession for him, everything poured out of him and the priest was there to listen and to give solace (2:508).

It is an enduring ideal for K.O. to repeatedly choose not to turn his back on the memories of the father. In his image of a childhood filled with mixed emotions and unhealthy relationships, K.O. holds on to the occasional warm memories he explores about the father. For instance, how the father becomes a totally different person when the grandmother visits (1:112–113) and when K.O. spends time with the father outside the suppressing boundaries of the home, for example, when they visited a record store and the father gave him credit for his taste of music (3:230). This and similar situations are meaningful for K.O. because he feels that even though they do not talk so much, it is important for him that his father notices him and his interests, that is, that he likes music. After the father has died, K.O. reflects on his occasional warm memories of the father and tries to define the father’s problem (6:373). However, despite the warm memories K.O. evokes, he cannot help admitting to himself that the father did not just fail his own son but in some ways even humanity; it was practically impossible for him to embrace the deeds of a good person. He remembers the father becoming instantly furious for the pettiest of reasons (2:338–339; 1:18–27) and the violence he suffers spans through the scale of physical slaps (2:338–339) and verbal mocking (1:44–45) to complete ignorance or disdain (3:38–41). Consequently, in his striving to acknowledge the father from a life perspective, he spends a lot of time and energy to process his feelings of being inferior to other people (6:938).

Channeling his fear into writing accomplishments (1:398), rather than letting them infect his relations with other people, becomes one of the great means for K.O. to be at peace with whom the father was and what became his destiny. As an adult, he is motivated to approach and enter the room of contradiction and mixed emotions about the father (1:301–303). In that environment of his equivocal images of the father, K.O. can explore a liberating space to express himself in words and endure the fear. How K.O. creates the necessary environment for living with the fear of the father is also visible in his childhood when he learns to live in different spaces and contexts. He spends a lot of time in the forest playing with friends and exploring the boundaries of the unallowable, he visits other families, observing and relating to his own conditions. These experiences of other environments and other person’s lives become an important means to counterbalance the fear of the father. He also finds an important resting place in the trusty communication with his mother and his brother, which helps him handle the unreliable father (4:160–161). Finally, his capability to be stoical is visible in this category as well. Suffering under a house-arrest, because of some insignificant offense of his, he draws the conclusion that it does not make any great difference to “ . . . the various phases which usually constituted an evening” (3:50).

#### Drinking

In his young years (1:74–76; 4:356–357) and up through his adult life, K.O.’s ideal of drinking is a state in which he experiences the joy and freedom of being carefree and without responsibilities. If alcohol has only primarily had that effect on him, he would be drinking all the time. Connected to the bare pleasure of drinking is also the experience that he is able to put an end to his loneliness (4:114–115) and come closer to the person he wants to be (4:356–357). In following his ideals of drinking, it is possible for K.O. to inhabit certain environments. In this case, to talk with a person he regards as a fellow intellectual (2:210). In spite of drinking being meaningful for K.O. in this and various other situations, he has major concerns about how the drinking is followed by anxiety. For example, he experiences huge memory loss in relation to heavy drinking, and he is not always able to reach his ideal state, where he is thriving and trouble-free, through drinking (2:187; 2:236).

K.O.’s experience of drinking as a liberating suspension of the dull and boring everyday life starts to disrupt. He finally reaches a limit where it becomes a distinct identity problem (4:403–404). The distinctions between the drinking environments and the everyday environments begin to fade. In his everyday nondrinking life—that is an easier living, for sure—he begins to suffer from the drinking events by experiencing direct feelings of guilt and disgrace (4:403–404).

Another concern related to drinking that deeply engages K.O. is how he relates to his father. Arriving home, after a football camp in Denmark where he has enjoyed all the pleasures of drinking at every level, he finds out that his father is having a party. He joins the party and can feel some comfort in continuing his drinking, but, at the same time, he finds it problematic to encounter the drunken father (1:192–206). This episode accentuates the dimension of K.O.’s problem that alcohol consumption deeply transforms the persona. Meeting his father at the party was an unpleasant experience where his father had turned from “hard” to “soft” in an unexpected way. The tacit knowledge, the roles, and the subtle cues upon which communication is built can be suspended quickly and old habits give no support. Instead, when the father drinks, K.O. needs to rearrange the world and get ready to meet a totally different person (1:202–203). By environing the different conditions of drinking, K.O. gradually realizes that the pleasurable transformation of the persona that ultimately follows from drinking might develop into lasting habits of a different behavior. When he experiences that his father, of all people, can be subject to such self-transformation, he takes it as a scaring reminder of the effects of alcohol in a life perspective. That is, it can function as a liberating as well as a devastating factor in life.

Worried about walking in his father’s footsteps, K.O. reads the father’s diary after the father has passed away (4:179–180). Even in this decadent period of the father’s life, he meets a very disciplined father who documented the names of everyone he met and the amount of alcohol he drank. K.O. is fascinated by the fact that the father seemed to be so aware of his own unhealthy habits and he realizes that to simply be aware of the proportions of the drinking is inadequate, because apparently that did not stop his father from ruining his life. Instead, what motivates K.O. in his own drinking is to handle it within socially accepted norms and to keep it to a limit where it makes him outgoing and talkative. That is, he needs to define for himself what kind of means the drinking is. For example, this is apparent through the conflicts he experiences when his drinking does not lead to any culturally approved outcomes. At a 30th party, K.O. become heavily drunk, and while he means that is the whole idea with the party, Linda finds him extremely embarrassing (2:287–288). Again, a healthy relation to alcohol is not necessarily to understand the physical harm is does, but rather to practice the drinking and coordinate it as a means to a culturally approved outcome. Thus, he is not really motivated to stop the drinking, but rather to understand the function alcohol has had in his own family and how to handle his role as an adult in a drinking context.

The main resources K.O. uses while drinking are his outgoing capacities and ability to suppress his feelings of alienation. The following description includes a drinking fellowship with his grandmother and the transformation of the consolidated norms in his father’s parental home. It is only a few days since the father passed away in his parental home and K.O. and his brother are there to clean up and take care of a drinking and senile grandmother who has been left in the ruins. On one occasion, he sits down and forces himself to share a drink with his grandmother and his brother (1:443–444). In his childhood, this house was careful to prevent others from prying, but now, they shared drinks in the middle of the day. This is a totally awkward situation, to sit in the disorganized living room and the dirty ruins of his father and grandmother’s life which is transformed into an experience of laughing relief when the remnants of the father’s norms are materialized through a bottle of alcohol in a brightly lit window. K.O. succeeds in his drinking when alcohol has this power to release tension and he can become the participant he needs to be; the brilliant intellectual, the forgiving son, the socializing adult, the son that handles the ruined house and a senile grandmother in the days following after his father’s death. In this way, drinking can function as a means for him as long as it supports *a different behavior and not a radical transformation of the persona*. The most definite sign of failure to use this means is exclusion and the incapability to fulfill the duties of a good father and husband.

### Belonging

In this second main theme of our analysis, K.O. struggles with feelings of belonging and alienation and has concerns about his needs and desires, possibilities and limitations, authority and mandate to be a part of different relations, groups, and arenas. In the following passage, he tries to mirror himself in the struggle of another famous Norwegian author:

No, in the new image I was drawing of myself there was also courage and backbone. Look people straight in the eye, say what I stood for. I had become more and more hunched, you see, I wanted to occupy less and less space, and on the island I began to straighten my back, quite literally. No joking. At the same time I read Hauge’s diaries. All 3,000 pages. It was an enormous consolation.

He went through worse times, didn’t he?

He certainly did. But that wasn’t the point. He fought *without cease* for the same, for the ideal of how he should be, as compared with the person he was. The determination to fight was extraordinarily strong in him. And that in a man who didn’t really do anything, didn’t really experience anything, just read, wrote and fought his inner struggle on a stupid little farm by a stupid little fjord in a stupid little country on the margin of the world. (2:556)

In the first theme, we saw that fatherhood became meaningful for K.O. when he approached it as a duty and a balance between closeness and distance. Here, the duty is extended to also include a continuous creation of a respectful self that includes the balance between K.O. the writer and K.O. the social person. In a life perspective, this is a progress in which he needs to dedicate himself to work on alternating identities. A good example of this awareness of how identity alters through life is when he reflects on the possibilities of having different names through life in relation to how identity and self-esteem alter (3:7). K.O.’s personal quest of who he wants and needs to be becomes visible through his concerns with authorship, to socialize and finally through the habit of smoking. These three subcategories highlight processes of belonging at different levels.

#### Authorship

K.O.’s authorship is closely related to how he is able to occupy different spaces. In his young years, reading books is meaningful to him because he can thereby belong to a world of his own. In this way, reading is a resource to live a good life. However, like becoming an experienced reader, he also makes his first experiences about the borders and boundaries between that world and the everyday life with friends. He worships literary heroes like Joseph Conrad and characters like Dracula (1:48), in contrast to action heroes such as Superman and Batman, but at the same time, he desires recognition in the culture of his own generation and class. He gives thorough descriptions about experiencing such gaps (3:304–307) between personal ideals and practical deeds when he enters the literary world and how it puts him in indeterminate situations (3:392). During many years, reading is K.O.’s ideal environment, and in this situation, he refers to all that he knows about winning and fighting through reading books and that he is unable to coordinate his literary wisdom with his experiences of losing. Later in life, trying to define himself as a writer, the problem tends to be the opposite; the wisdom of living an empirical life is not successfully transformed into literary narratives (5:188).

As a student at the writer’s academy, he defines his struggle with this gap. Idealistically, for him, the expressed art and the lived life connect; this is a key component in living a good life. Reading, as well as writing, becomes a problematic experience for K.O., and he struggles to find his own style. To be a good writer is not always about skills, he notes, but also about understanding literature in a culturally approved way (5:241–243) and to develop a unique style (5:158–160). Apart from the problem of understanding literature in the culturally approved way and to find an artistic expression of his own empirical life, one of his biggest challenges according to occupying different spaces is referred to as being able to sustain in qualitative experiences of emotional conflict. This often means that he needs to put himself at risk, even his personal self. According to K.O., writing a text with high aesthetic quality means writing a text with a soul in it—his soul (4:506).

K.O. feels a desire to be comfortably at home in all places/environments and the engagement in his authorship is, to a great extent, expressed through the need to occupy a place where he, as an artist, can dwell and use intimate material from the private world in a mature and brave way without being emotionally trapped and locked for artistic expression (6:65). Again, the work to explore the artistic environment is directly associated with the risk of losing the private self. But, it is a risk worth taking to acquire a good authorship (6:976). One of the important means to sustain in qualitative experiences of emotional conflicts without losing the self or letting the writing experiences invade family life is the ability to develop certain habits. K.O. creates a living where routines are cornerstones to maintain focused writing. These habits are important for a working life which promotes health (in terms of avoiding anxiety and doubt) and progress (5:608). Although he finds it problematic to experience the gaps between the literary world and the lived life (regardless of what direction), he still believes in borders between them. This becomes very clear when he tries to juggle family obligations and professional obligations (1:39); the private and the artistic selves cannot become one.

As a renowned writer, the ideal for him is still that expressed art and lived life connect, but with a more solid belief in a cumberless motion between an artistic world (e.g., alone in his studio) and all the rest of the everyday duties and pleasures of life. Reading is still important to him, but more as a way to reside in his own world amid everyday activities (2:267–270), or to inspire him to write honestly and clearly. When he begins to identify himself as a writer more than an experienced reader (as in his young years), the reading becomes more of a resource to him, rather than an idealistic environment on its own. The reading can spark the writing (4:428–429).

Another resource for K.O. to live a good life as an author is the audience. That is, it is not satisfying enough to only occupy an artistic private sphere. He needs a collegial environment, too, to consummate his artistic progress (4:30). Authorship is about belonging to different worlds and to be able to alternate between these. That is not to be seen as a back-and-forth movement in which he is opening one door and closing another. Rather, it is about a continuous environing of the conditions he encounters. He feels a need to connect the personal empirical life to his literary life in artistically satisfying ways. He knows he has succeeded when he has been able to coordinate vague memories and a feeling of the childhood into a biographical narrative within a specific context.

#### To socialize

During the years when K.O. wrote *My Struggle*, his close friendship with Geir is the center of his social life. They talk on a daily basis and K.O. has an intense feeling that he can be himself when talking to Geir. He is comfortable and at peace with himself, thinks freely (6:223–224), and has a strong feeling that Geir finds him interesting (6:240–241). To acquire the ideal relationship that he has with Geir, and that he also sometimes experiences with his wife and children (4:242; 5:556–558), is a struggle for him in the social life outside this space. He wants to move freely between environments and live a life of qualitative experiences.

K.O. has many concerns about the limitations of, and possibilities to, suspend meta-level reflection (e.g., ideology) to “just live” (6:247–248; 1:297). Again, a life expressed as a *lived life* is an ideal for him, at the social level as well as in the artistic creation he aims at in his authorship. Already in his young years, his reading and music listening open up doors to a different space for him where he can experience things in this direct way (3:376). To experience life in this direct and intense way becomes a certain style for him too and the feeling of being different to his classmates pervades his experiences. He is often accused of being feminine (3:443–445) and too aware of his clothes and taste in music. One example of this is that he strategically picks songs from “Klasstoppen” to distinguish himself as an outsider (3:392). Although in many situations he is taking pride in being different, to some extent, he also does what he must to be a child among others. For example, he prepares a favorite boats-mopeds-cars-list in case someone asks him about those things (3:383).

The fear of exclusion or to experience actual exclusion affects him deeply sometimes, for example, when he is rejected by his grandmother and banned from her house. His mother tells him that his grandmother called:

Yes. It wasn’t a pleasant conversation, I’m afraid. She said . . . well, she said you weren’t to go there any more. She said you’d never have anything to eat whenever you turned up, you were shabbily dressed and were always asking them for money. (4:265)

K.O. is devastated about what his grandmother said about him and burst into tears. He cries, not only because he does not fit in but also because he has been deprived his membership of that space. Later, in his adult life, he finds social life ideal in small and close contexts of intimacy where those involved are in mutual understanding and experience the freedom of communication.

Indeterminate situations become explicit at a social level through his ambiguous feelings of seeking comfort in being an outsider and at the same time desiring a broad acknowledgment for the qualities he possess (e.g., his taste in music, his intellect, his storytelling, his norms as a parent). At a psychological level, we can identify an ambiguous feeling is his longing for an outgoing personality and that, at the same time, he identifies himself as an introvert person, for example, by mirroring himself in introvert famous sportsmen (3:383) and intellectuals. In contrast to his father, who was socially skillful but never a member of the social life, K.O. wants to be a full member. One thing that he experiences as problematic in connection with such a membership is that his capacity to laugh becomes an utterly private expression for him. It is a technical skill that he knows he can perform but never fully enjoys in the company of others. Instead, he smiles (5:76).

Not being able to laugh in social situations is related to a need to practice his ability to express happiness and pleasure among other people. Although in similar ways, he experiences social life awkwardly, many times he has a strong winning mentality and an eagerness to demonstrate intellectual brilliancy (5:70–71) that motivates him to take part in social life. He wants to be more outgoing and real and, indeed, being more comfortable in approaching women (4:85–86, 4:392–393). Many of these uncertainties in the social life have to do with how he wants to be capable of bringing his “self” from one place to another (2:74–75).

In bringing his self from one space to another, he notes, and becomes worried about how he habitually changes not only personality but also values and ideals when moving between the personal and the social worlds. Maybe this is most explicit in the reflections of his conduct when working with disabled people. In the actual situation, he disdains them deeply even though he knows that he is supposed to feel sympathy (5:354–355). He is pained by guilt even when meeting strangers or acquaintances walking down the street (2:149–150). He describes it as a feeling of being inferior to other people in social situations (6:885) and that he has a strong desire to adjust to social situations (2:348).

Even when getting used to situations and being adaptable, which is his ultimate method to handle many social situations, he experiences a deterioration in his character. His means to participate successfully in social life are often of an instrumental character, like when he draws on his ability to smoke, drink, and be stoical (5:170–171). Closest to his ideal of moving without friction between the many spaces of social life, expressing and experiencing the world together with fellow beings, are the events when he is able to connect to the Divine.

#### Smoking

The weeks after the birth of their first child, K.O. describes the euphorical feeling he lives through, that he becomes absorbed in his writing and it becomes everything for him:

I moved into the office, wrote day and night, sleeping an hour here and there. I was filled with an absolutely fantastic feeling, a kind of light burned within me, not hot and consuming but cold and clear and shining. At night I took a cup of coffee with me and sat down on the bench outside the hospital to smoke, the streets around me were quit, and I could hardly sit still, so great was my happiness. Everything was possible, everything made sense. (2:79)

Ultimately, K.O.’s smoking habits allow him to alternate between an artistic state in which he organizes and creates his environment into an everyday state where he can be a person among others who experiences the world directly (6:83).

K.O.’s private life is a hectic bricolage in deep contrast to his professional life that is ordered and creative although filled with hard and strong feelings. Smoking often establishes a floodgate between these two environments. In the several smoking situations in K.O., he sits on a bench or on the balcony watching the traffic, noting the shifting weather conditions or just rests in his stream of thoughts. In doing that, the smoking becomes a routine that supports his working day with a certain rhythm, that is, idealistically he can make transfers between the private and the artistic sphere but still keep the continuity of being the very person who experiences and grabs the empirical world. However, smoking is not only a functional habit that works as a basis for his daily rhythm moving between environments, but it is also connected to certain problems. In his young years, smoking relates to another life and is very unwelcomed by his father (3:454–455).

As an adult, smoking becomes a problem for him when he tries to uphold it in his role of being a father and he experiences ambivalence about the decision to stop. Linda, his wife, is asking him to stop (6:911–912), but K.O. continues to smoke. Despite the obvious problems it entails as a means for him to make certain transfers, for example, he smokes before entering the private world and dares read the email answers from his uncle about some passages in his book (6:130). He also uses smoking to cope with his anger about his mother’s and brother’s moderate excitement over the fact that Linda and he are going to be parents for the first time. He lights up another cigarette and spends some time in the yard before going back to his study:

I lit another cigarette and noticed I was not completely satisfied with their reactions. We were going to have a BABY, for Christ’s sake! This was an ENORMOUS event! (2:304)

In his young years, smoking is a tool to acquire mobility between his outsider world and the world of his ordinary friends:

[W]here should I go? Where should I stand? I could sit in the library and read, or sit in the classroom and pretend to be going through homework, but that was tantamount to signalling I was one of the outsiders and was no good in the long run, so in October that year I started smoking. (1:48)

This description of how he saw the benefits of smoking is a description of how he is able to create a small manageable social space through smoking. Again, in his young years, sitting at his grandparent’s house (many years later after he was banned there), he overcomes his own stigma about the cigarettes and picks them up in front of the grandmother (4:189).

Even though smoking becomes a technique to inhabit environments that he finds awkward (e.g., in the young years) and supports his work day with a certain rhythm in that he gets dressed (for work) and undressed (to meet the private) by means of his smoking habits, there is always a lurking stigma of an irresponsible father and husband.

## Discussion

In our analysis of *My Struggle*, we have sought to understand health through salutogenic descriptions of the endeavors and complexities of everyday life. We have thus not only recognized health behavior as a chain of activities but have also tried to analytically depict the transactional relationships between a person’s roles (father, son, husband, author), experienced obstacles (fear of the father, to socialize), and habits (drinking, smoking). Against this background, it is evident that understanding health as experience is not about following how one action leads to the next (van Woerkum & Bouwman, 2012), but rather describes how a person makes transpositions between different identities, relationships, places, and experiences in everyday life. In contrast to more epidemiological studies, the contribution of qualitative health research is to further knowledge and understanding about the central mechanisms of unhealthy behavior and the key determinates for successful health promotion interventions (Commission on Social Determinants of Health, 2008). Hence, the starting point of this study is a general question about how empirically feasible it is to uncover the reciprocal relationships between different mechanisms and how this may or may not contribute to individuals’ health in their lives *as a whole*.

This has been tested using a unique autobiographical narration as empirical data for analyzing health as experience. The salutogenic informed research questions (McCuaig and Quennerstedt, 2018), together with Dewey’s theory of environing, have enabled us to identify the underlying regimes in K.O.’s life story. The methodological addition to salutogenic research and the narrative approaches can be concluded in a three-step procedure. First, they can reveal commonplace themes stated in biographical material, which include approaching life stories from the position of an everyday reader or listener. Second, the identification of commonplace themes can help to establish analytical starting points for working through salutogenic informed research questions. Third, salutogenic questions must be operationalized (as shown in the methodological section) in ways that both adapt to the unique biographical material and create reliable data. In following this procedure, our study has illustrated how data can be organized so that ideals, problems, means, and resources hang together (a terminology theoretically informed by Dewey’s theory of environing) in tiny tales (our own methodological construct). In *My Struggle*, K.O. identifies his life themes in the following way: “A life is simple to understand, the elements that determine it are few. In mine there were two. My father and the fact that I had never belonged anywhere” (Knausgaard, 2014a, p. 628). In using these life themes as an analytical starting point, we have identified *paternity* and *belonging* as “regimes” and presented “tiny tales” about how ideals, problems, means, and resources hang together in K.O.’s narration habits (the subcategories). The biographical material was strategically selected because it included a functional coordination of two notable physiologically unhealthy habits (drinking and smoking). In so doing, we strove to secure a level of empirical resistance in validating the method and explored what happens when physiological health and social health are placed at the same analytical level. The importance of taking a broader perspective on, for example, drinking behavior, is stressed by Pedersen et al. (2019). In their study, the participants—men with alcohol problems—experienced health professionals as exclusively focusing on risky health behavior, rather than taking their entire life situations into account as they had hoped.

Salutogenic researchers have drawn attention to the difficulties of adopting a truly salutogenic perspective on health (McCuaig & Quennerstedt, 2018; Suominen & Lindstrom, 2008; Super et al., 2015). As both researchers and practitioners, we are intuitively inclined to understand health as a state in which we are freed from every aspect of illness or disease and that disease cannot include any dimensions of well-being or health. Such tacit assumptions have a huge influence on how researchers inquire into human beings’ experiences of health and how practitioners organize and carry out health behavior counseling with clients. In operationalizing Antonovsky’s health theory, McCuaig and Quennerstedt (2018) have therefore suggested a collection of salutogenic informed interview questions that potentially dissolve pathogenic foundations and instead promote dialogue about “a good life.” Sidestepping what might be obvious questions about “good health,” these particular questions encourage narratives that can facilitate the identification of underlying regimes constituting what certain groups or individuals respect and value about their lives.

One important methodological result of our study is that the empirical strategy for understanding such regimes makes it possible to position physiological health and sociological health at the same analytical level. Thus, in this study, the analytical focus on “a good life” has resulted in the identification of “belonging” as an underlying regime in K.O.’s narrative—a regime that constitutes the function of the smoking habits in his life as a whole. For example, K.O.’s inability to socialize became a problem for him in high school (1:48). His immediate feeling of alienation was strongest during the breaks when he did not know how to connect with or approach other students. To handle the disruption of the comforting environment of the lessons, smoking is narrated as the primary vehicle of transposition between lesson and break. In the smoking area, his social presence was not justified by style or communicative skills, but rather through the humble maneuver of lighting up a cigarette. That technique was enough to make him feel as though he belonged. Moreover, the analysis showed that K.O. used that embodied habit of environing the social space in different phases of his life. For instance, it became an important resource when he as a renowned author invited friends to his home for dinner (2:344–345). Leaving the table and going onto the balcony for a smoke was not just an escape, but also an invitation to others to share his space. The physiological unhealthy habit of smoking thus had *an embedded function of expanding the social belonging*—and increased health in that particular situation—for K.O. In this way, the use of methodological tools that can identify underlying regimes—in this case belonging—can facilitate a deeper understanding of the meaning of smoking in a life context. Studies that make use of the suggested method can complement other studies of, for example, men’s smoking habits, where the difference between smoking at work and smoking at home are identified (Oliffe et al., 2010). Indeed, it is also important information when conducting interviews about smoking or engaging in health behavior counseling with clients in that it takes all the different roles, obstacles, and habits in our lives into account.

### Concluding Remarks

Instead of using salutogenic research questions as tools for conducting research interviews, we have tailored them into a methodological framework that facilitates investigation of the transactions between organisms and their environments, that is, the swimmer and the river. This has been made possible through Dewey’s meaning theory and an account of how human beings environ their circumstances in the context of inquiry. Various studies have used transactional perspectives to investigate how individuals and environments are in mutual relationship with each other and how this relationship relates to the habits and outcomes of sport pedagogics (Andersson & Maivorsdotter, 2017). We have furthered these studies by employing a health analysis of the transactional details of the significant regimes in K.O.’s life. We have focused the analytical spotlight on the more shadowy corners of everyday health experiences by explaining how these regimes gain momentum through the process by which individual actions and environmental conditions both affect and adapt to each other in the course of life.

However, there are limitations with this approach that are worth noting. Asking broad questions about what a good life is often leads to stories with an overwhelming richness of detail. The suggested method is therefore time-consuming and the research questions sometimes need to be adjusted during the data collection and/or data analysis. This is also an explorative study design that is not suitable for every research project.

Despite these limitations, the general contribution of this study to the field of health research is a framework that can account for individuals’ salutogenic experiences of health, can guide salutogenic informed questions, and allows us to problematize to who and whom such questions should be addressed. The choice to employ this framework on a professional author’s autobiographical story is motivated by Brinkmann’s (2009) claim of the author as a researcher of social practice. He argues that great authors distinguish themselves as studious observers of daily life and execute sophisticated methods that are capable of capturing experiences and underlying trends in society that reach beyond the subjective and personal domain of living. The expertise of great authors is not, for example, their parental wisdom, or their execution of bad habits (drinking, smoking), but rather their highly cultivated ability to express life from different aspects and to connect to events and experiences that we can all relate to in our own unique ways. All the details of our insignificant daily endeavors can be worthwhile in a larger and more circumstantial context in that they articulate what we sometimes find impossible to describe. Getting to know ourselves and others better through the relationships of which we are part (e.g., parenthood, alcohol, smoking) is important for both researchers and practitioners. However, as it is not only professional authors who create stories for such purposes, the methodology outlined in this article should also be tested on other life stories told off- or online by “unprofessional” storytellers.

## Notes

1. All the books by Knausgaard (2014a, 2014b, 2014c, 2015, 2016, 2018) are referred in the text in the form of (3:234), where the findings refers to volume 3, page 234.
